# Surgical Cases

**Published:** 1858-06

**Authors:** C. Brackett

**Affiliations:** Rochester, Fulton Co., Indiana


THE CHICAGO

MEDICAL JOURNAL.

VOL. I. JUNE, 1858. No. 6.

### ORIGINAL COMMUNICATIONS.

ARTICLE I.

### SURGICAL CASES.

BY C. BRACKETT, M. D.

ROCHESTER, FULTON CO., INDIANA, APRIL 30TH, 1858.

EDITORS OF CHICAGO MEDICAL JOURNAL,—I notice in the April No. of the *Journal* (page 191), that you have an article post-marked "Rochester, Indiana," wanting a signature. I sent several articles, a part of which only have appeared. You will know my writing by this. If they are alike, use my name. I can tell if the article is mine when I see it, and if it should prove not to be mine, I can correct it. I supposed the reason of their not being published all was that they lacked interest.

I sent, a few years ago, the details of a case of ascites, which I thought interesting, but never heard from it, so supposed it was not considered worthy of publication. I thought it of interest, from the fact, that the patient recovered, and that I had taken at different times extraordinarily large quantities of fluid from her; as, at one tapping, one hundred and twelve pints, at another, ninety-six, etc. For the past five years, the patient is hearty. This, with several other cases of dropsy, I will send, if you think they are worth publishing. I send herewith some cases of injuries of joints.

CASE 1. Louisa E. Brackett, aged 6 years, last November, knelt down to pull out the lower drawer of a bureau. A No. 6 needle was sticking point foremost in the carpet; she knelt on it, and it entered the knee joint of right leg; entered the femur on a line below, and to the inside of the central line of the patella. On extending the leg, the needle broke off, leaving about the fourth of an inch of the head in the femur. The other piece I removed a few minutes after the accident. Treated the case by perfect rest to the joint, maintained by splints, *opiates*, or rather opium, *as often, and as much as necessary to procure perfect rest*; laxatives as necessary; tinct. iod. sat. as local application, and cloths wet with spirit lotion. After six weeks, she could begin to touch the foot to ground, and swelling began to abate; thenceforward the swelling abated rapidly, and the joint resumed its functions perfectly.

CASE 2. Miss Marine, of Gilead, Miami Co., a few weeks since, ran a No. 6 needle point foremost into the knee joint of right leg when knee flexed. Needle entered from above, pierced the cartilaginous portion of inferior end of femur, internal to patella, was driven in out of sight. I saw her three weeks and five days after the accident. Knee had suppurated and opened, each side discharged freely; treatment had been antiphlogistic anodynes, *sparingly* used.

I made an opening at the point of puncture, found the head of needle one quarter inch below surface, removed it with some little trouble. Needle was *bent* like a fish-hook, caused, I suppose, from point of needle having entered the upper articular surface of tibia, and having been turned up by extending the limb after the accident. Patient is doing well.

CASE 3. *April* 9, 1858. John Wilson, aged 11, was struck on external ankle by stick of timber to which horses were attached. Tibia was burst through integuments of inner ankle, making wound half of circumference of the limb. The tibia ploughed the ground about four feet before boy was extricated. Drs. Cleland and Gould cleaned the wound, which was firmly impacted with dirt and straw, returned the tibia, deprived of its lower articular surface, to its place, and the attempt to save the limb is so far successful, that when I saw the boy last, day before yesterday, he was sitting up, with starched bandage to limb, comfortable. Discharge from wound lessening, healthy. Boy in good spirits, appetite good. Treatment has been opiates *as often and so much as to allay, perfectly, pain and spasm,* with perfect *rest* of injured joint.

CASE 4. Mark M——, aged about 20, shot, with a rifle ball, through metatarso phalangeal articulation from above down; removed all splinters of bone that I could find, patching, etc. Treated by *opiates as often and much as necessary to relieve, perfectly, pain and spasm;* perfect *rest* of affected part; cure perfect and speedy, without a bad symptom except discharge of some spiculæ of bones which were detached during the healing process.

CASE 5. John Carter, aged 18, June, 1856, shot, with a rifle ball, through centre of humerus, above junction of upper third with lower two thirds. Six weeks after accident I saw him first, humerus (tightly bound to rough splint), elevated above the level of the joint; wound discharging sparingly, depots of matter about the joint and above the clavicle, hectic fever, no appetite, erysipelatous inflammation of arm, anterior and posterior sides of chest, tumefaction, and much emaciation, and amphipneuma. Has been treated with *antiphlogistics.* I brought arm down to side. Treated with *opium* and *quinine, as much as he can bear;* generous diet; began to improve rapidly, and discharged perfectly cured, or perfectly well.

CASE 6. Called to Mrs. VanMetre, seventeen miles S. W. of Rochester, last week; sent Dr. Gould. Case: dislocat. jaw of eight and forty hours' duration. Two of the Smiths, and some others, had been at it at the time; had broken out three teeth and skinned the jaw in divers places, and at last concluded it could not be reduced by force, but *a liniment could be applied that would draw it in* in *eight* hours. Dr. Gould reduced it without difficulty, as the patient was unwilling to wait for the operation of the liniment of the Smiths, or for a preparation of lobelia which a steamer said was infallible. A case of this kind betrays the ignorance of such vile pretenders; but, what thousands of lives are lost, and what weeks of suffering endured by the sick, through their ministrations.

CASE 7. —— Perry, aged about 36. Both hands blown off by premature discharge of cannon. Assisted by Drs. Howes, Robbins and Mann, removed remnants of carpus. Second day, patient out on street; recovered without bad symptom. Treatment, *opium and stimulants ad libitum;* recovery perfect, without bad symptom. Had served in Texan Revolution and Mexican War; had had fractured skull from sabre wound, received in Texan Revolution, and done well through all his troubles.

The case of ununited fracture of femur which I reported in last December No. of the Journal, has terminated in the perfect union; limb strong. From this time on, we need not be discouraged, for nature will do wonders in the cure of injuries, if *she has a fair chance.* Give her *rest* and *quiet.*

The case of paraphymosis, reported by me in a former No., terminated by formation of urinary fistula which can be healed; yet I think my undertaking to save the penis from amputation, was of doubtful benefit to the patient; for, without doubt, a short member well rigged, is preferable to a wife, to one of full length, wanting fore-mizen and bobstays.

*March* 17*th*, 1853. Mrs. Moore applied for removal of cancer from face, occupying the space from just below the ear to near the point of the chin, two inches broad in the center; open, and discharging offensive matter, attended with lancinating pains. Patient unable to open the mouth; can only take a fluid diet sucked through a tube. Disease of some twelve years' duration, but only for past year seriously troublesome. Eight years previously I saw the case, then only a tumor movable in left cheek of size of a hazel nut, and on account of occasional sharp pains through it, I advised its excision; but she remarked to me thus: "No, no, I tell thee, Charles, when I die I want to go altogether." Now unable to speak, she and her friends are anxious for its removal, even if only for temporary benefit. Assisted by Drs. Hagins and Hoover, I removed all the diseased parts. Her condition was bettered every way until the wound healed, when it again appeared from the parotid gland, involving gradually all parts concerned in deglutition, when she died of starvation.

During the summer of 1852, James Carter, aged 50, came to me with what I thought to be a cancerous disease. On the chin, directly in the center, was a tumor of florid red color, size of hickory nut, open in center, attended by sharp lancinating pains; tumor quite hard, boundaries not defined; countenance sallow, cadaverous, general health poor. With little thought of benefiting him, I commenced using the chloride of zinc in substance to the tumor, holding it to the spot till dissolved with a bit of paper. Gave him carb. fer. internally, and directed him to keep bowels regular. Applied the caustic every other day, or at longer intervals of two, three and four days. Tumor decreased with each application, and disappeared. General health became good, and to this time there is no appearance of a return, or tendency to return.

Mrs. Kiever, on Eel River, Miami Co., sent for me to remove breast, which was cancerous. In company with Dr. T. H. Howes, of this place, I went over, and found the patient with large melanoid open cancerous ulcer, deeply excavated, and discharging exceedingly offensive matter; ulcer seven inches in diameter. Assisted by the Doctor and others present, I carefully removed every vestige of diseased tissues, going far up in the axilla for all parts with hardened or unhealthy feel. The patient recovered, and for five years after had good health, as I have heard; since which time, I have not heard from her.

Mrs. Fischer, of Kosciusko Co., came to me to have excised what the "*cancer doctors*" had told her was an *eating cancer*. Tumor hard on left cheek, reaching to lower edge of lower jaw; ulcerated in center, hard, red, and somewhat painful all the time; immovable. I removed two carious teeth from lower jaw of that side; gave a box of simple cerate, which they thought was "cancer salve," and told them to return in two weeks if face not better. After a few weeks, saw her husband, who said the face was about well. In a short time she was free from what she and her friends thought a "horrid cancer."

H. Brunson, aged about 50, came to me from South Bend, St. Joseph Co., with hæmorrhoidal tumor of twelve or fifteen years' duration. Tumors unhealthy in appearance, occupying whole circumference of rectum; patient sallow, anæmic, suffering much from pain and loss of blood. Proceeded, assisted by Dr. Gould, to ligate the tumors; passed double threads both ways through the mass as high up as possible; tied in this way four different points; drew the ligatures as tightly as possible, relieving pain by opiates and chloroform; applied cold water dressings. In about eight days, parts of tumors commenced falling off. Applied new ligatures in about ten days to parts not first ligated. During the cure, before the ligatures were all off, he pulled at one so hard as to tear off a vessel, which bled a stream of the size of a goose-quill; he was almost completely drained of blood before I found it out; fainted, or nearly so, several times; rest and pressure checked the hemorrhage. After four weeks' treatment, he was discharged cured, though still extremely debilitated from loss of blood. There was one part of the tumor through which the ligature would not cut. To this I applied the chlor. zinci with best results. Some few months afterward I heard that my patient died suddenly while seated in his chair. This, I suppose, was from rupture of aneurismal aorta, though I did not hear the particulars.

*May* 3*d*, 1858. The following case is interesting in some particulars: Miss Gangwehr, aged 18, was thrown from her horse, and injured in the humerus. She was treated for fracture; humerus bandaged and splinted; forearm put in a sling, and hand allowed to hang down prone. After the lapse of some weeks, the bandage and splints were removed, but the hand and forearm were useless, that is, she had no control over the extensors of the arm, and presented the appearance of one suffering from the "hand drop" of lead palsy. This state of things continued for three months, when her father consulted me. I treated the case as follows: extended the hand so as to bring the fingers as nearly at right angle with the wrist as possible, compatible with comfort to the patient; maintained it in that position by a splint made of, first, a copper clasp, two inches wide, embracing the forearm immediately below the elbow, another of zinc, of same width, for the wrist; these were connected by four wires soldered to the plates, to run down the outer face of the arm, and extending as low down as the ends of the fingers, with a branch to the thumb; at the ends were rests for fingers and thumb. These wires were so bent that they maintained the fingers and thumb as much extended as the patient could comfortably bear. This was worn day and night, only when it was taken off three times per day, to give the arm the cold *douche* and thorough friction after with a coarse towel impregnated with salt (by wetting in salt water and drying); after this, the arm was wet with the following wash:

| | | |
|---|---|---|
| ℞ | Strychnine, | grs. x. |
| | Acidum aceticum dilutum, | ℥ii. |

The splint was then reapplied. This treatment continued four weeks, and perfect cure the result.

During the time of bathing and friction, the hand was kept extended—not allowed to be flexed.

I consider that the continued extension of the flexors, and the chance thereby given to the weaker *extensors to retract and contract*, was the chief agent in the cure, as the continued elongation of the extensors and the contraction of the flexors (by the position maintained during the treatment for the fracture of the humerus), was the chief cause of the loss of power in the hand. For the treatment of the different forms of talipes (except calcaneus), this object should be kept in view, and attained by an apparatus on the same principle adapted to the leg, and should be worn *during the night* (if the patient is old enough to walk), and a Scarpa's shoe, or whatever one the surgeon prefers, can be worn during the day time. This will *expedite* and *perfect* the cure wonderfully; in truth, I do not believe that a cure will be *perfect* without it.

The application of this principle is original with me, or at least I do not recollect having seen it in practice except by myself. The first case to which I applied it was to a boy, —— Adams, twelve years since, who had talipes caninus. I divided the tendo-Achilles, brought up the foot, and made a cure that might be called nearly perfect; as, when he left my care, he could wear boots, and on the affected limb only one thickness of sole leather was requisite additional to the heel to render his walking perfect; and he left off my apparatus before I was willing, saying that he was well enough.

This thorough extension, maintained by the apparatus above described, varied to suit the case, renders the cure of hand drop and talipes a much more simple matter, and expedites the cure to a surprising extent. If I am in the dark in this matter, or only reiterating a common practice, I would like to know it from some of the older or abler members of the profession.

